# Proteomics profiling for the global and acetylated proteins of papillary thyroid cancers

**DOI:** 10.1186/s12953-023-00207-8

**Published:** 2023-04-26

**Authors:** Wei Wei, Yuezhang Wu, Dong-Dong Chen, Yuntao Song, Guohui Xu, Qi Shi, Xiao-Ping Dong

**Affiliations:** 1 Key Laboratory of Carcinogenesis and Translational Research (Ministry of Education), Head and Neck Surgery Department, Peking University Cancer Hospital & Institute, Beijing, 100142 China; 2 State Key Laboratory for Infectious Disease Prevention and Control, Collaborative Innovation Center for Diagnosis and Treatment of Infectious Diseases (Zhejiang University), National Institute for Viral Disease Control and Prevention, Chinese Center for Disease Control and Prevention, Chang-Bai Rd 155, Beijing, 102206 China

**Keywords:** Papillary thyroid cancer, Proteomics, Acetylated proteins, TMT labeling, KEGG

## Abstract

**Background:**

Papillary thyroid carcinoma (PTC) is the most common endocrine malignancy cancer among the malignancies of thyroid. Despite of wide usages of proteomics in PTC, the profile of acetylated proteins in PTC remains unsettled, which is helpful for understanding the carcinogenesis mechanism and identifying useful biomarkers for PTC.

**Methods:**

The surgically removed specimens of cancer tissues (Ca-T) and adjacent normal tissues (Ca-N) from 10 female patients pathological diagnosed as PTC (TNM stage III) were enrolled in the study. After preparing the pooled extracts of the whole proteins and the acetylated proteins from 10 cases, TMT labeling and LC/MS/MS methods were applied to the assays of global proteomics and acetylated proteomics separately. Bioinformatics analysis, including KEGG, gene ontology (GO) and hierarchical clustering were performed. Some differentially expressed proteins (DEPs) and differentially expressed acetylated proteins (DEAPs) were validated by individual Western blots.

**Results:**

Controlled with the normal tissues adjacent to the lesions, 147 out of 1923 identified proteins in tumor tissues were considered as DEPs in global proteomics, including 78 up-regulated and 69 down-regulated ones, while 57 out of 311 identified acetylated proteins in tumor tissues were DEAPs in acetylated proteomics, including 32 up-regulated and 25 down-regulated, respectively. The top 3 up- and down-regulated DEPs were fibronectin 1, KRT1B protein and chitinase-3-like protein 1, as well as keratin, type I cytoskeletal 16, A-gamma globin Osilo variant and Huntingtin interacting protein-1. The top 3 up- and down-regulated DEAPs were ribosomal protein L18a-like protein, alpha-1-acid glycoprotein 2 and eukaryotic peptide chain release factor GTP-binding subunit ERF3A, as well as trefoil factor 3, thyroglobulin and histone H2B. Functional GO annotation and KEGG pathway analysis based on the DEPs and DEAPs showed completely different changing pictures. Contrary to the top 10 up- and -down regulated DEPs, most of which were addressed in PTC and other types of carcinomas, changes of the majority DEAPs were not mentioned in the literatures.

**Conclusions:**

Taken the profiling of the global and acetylated proteomics together will provide more broad view of protein alterations on the carcinogenesis and new direction for selecting biomarker for diagnosis of PTC.

**Supplementary Information:**

The online version contains supplementary material available at 10.1186/s12953-023-00207-8.

## Background

Thyroid carcinoma is the most common endocrine cancer among the malignancies of head and neck [1–3], which accounts for about 1% of all kinds of carcinoma and comprises 91.5% of whole endocrine cancers. Recent statistics shows that the incidence of papillary thyroid carcinomas (PTC) is increasing in the past years and accounts for 80% of all thyroid cancers [4, 5]. Timely diagnosis and appropriate treatments are critical for raising long-term survival rate of PTC patients. Cytological examination and fine-needle aspiration, sonography, magnetic resonance imaging, and computed tomography have been used in diagnosing for PTC. The most effective test for distinguishing malignant from benign thyroid nodules is ultrasound-guided fine-needle aspiration biopsy which has approximately 93% sensitivity and 75% specificity. Advancing in the proteomics has introduced new techniques for screening the biomarkers and improving diagnosis of different kinds of cancers to a new horizon. Till now, some biomarkers for thyroid carcinoma diagnosis have been found including galectin-3 [6], fibronectin-1 [7], CITED-1 [8], HBME1, cytokeratin-19, TPO [9], etc., however, most of these biomarkers lacking specificity or having poor positive predictive value to some degree [6–9].

Protein acetylation in which the acetyl group transferring from acetyl-coenzyme A (Ac-CoA) to a specific site on a polypeptide chain is the major post-translational modifications (PTMs) in most eukaryotes. About 90% of proteins become co-translationally acetylated at their N-termini of the nascent polypeptide chains in humans. Till now, hundreds of different PTMs were identified including acetylated proteomics profiling with new techniques emerging and especially in the mass spectrometry field. Because of more consumption of Ac-CoA during acetylation and more consumption of NAD^+^ during deacetylation by specific KDACs (lysine deacetylase), acetylation process participates in the metabolic processes and energy homeostasis of body. Consequently, unbalanced acetylation machinery can lead to severe diseases, such as neurodegenerative diseases, cardiovascular disorders, cancers, etc. [10–12].

With the help of LC-MS/MS, we screened the global and the acetylated proteomics profiling of the tumor tissues from 10 PTC patients at TNM stage Ш, controlled with the normal tissues adjacent to the lesions from the same patients. Totally 147 out of 1923 identified proteins were considered as the differentially expressed protein (DEP), including 78 up-regulated and 69 down-regulated, respectively. Fifty-seven out of 311 identified acetylated proteins were differentially expressed acetylated proteins (DEAP), among them 32 were increased and 25 were decreased. Further, some bioinformatics analyses, such as hierarchical clustering, KEGG pathway, gene ontology (GO), based on DEPs and DEAPs were performed. We found that either involved GO processes or KEGG pathways were almost completely different between the global proteomics and acetylated proteomics.

## Methods

### Ethics statement

The research protocol was approved by the Ethic Committee of Peking University Cancer Hospital & Institute, and the subject signed the informed consent form approved by the Institutional Review Board at the Peking University Cancer Hospital & Institute.

### Samples of thyroid cancer

To address the possible difference in the protein and the acetylated protein expressions between the carcinoma tissues and the normal tissues, we enrolled the specimens of the surgically removed tumor tissues and the normal tissues adjacent to the lesion sites without abnormal pathological signs from 10 patients of PTC. Approximately 700 to 800 mg specimen of each patient was obtained, 200 mg for global proteomics and 500 mg for acetylated proteomics profiling assay. All enrolled samples were pathologically confirmed by the pathologists in Peking University Cancer Hospital and Institute, excluding the evidence of other chronic thyroid diseases, such as chronic lymphocytic thyroiditis and autoimmune thyroid disease. As more numbers of female patients of PTC than male patients, all enrolled patients were female in this study, to eliminate the potential influence of gender. The ages of those 10 patients ranged from 56 to 83 years old, with the average of 65.6 year-old. All enrolled patients were at TNM stage III. The ages and TNM stages of those cases were summarized in Supplemental Table 1.

### Protein extraction

Tissues were homogenized in the lysis buffer containing 5 mM C_4_H_7_NaO_2,_ 2.5 mM NA_3_PO_4_, 1 mM Na_3_VO_4_, 8 M Urea and 30 mM HEPES. The lysates were mixed with 10% TCA-acetone and stored at -20℃ overnight, and then, collected by centrifuging at 4℃ for 30 min at 20,000 rpm and washed with acetone for three times. After adding lysis buffer, the preparations were exposed to ultrasonic under power 180W for 5 min. Next, after centrifugating for 30 min at 20,000 rpm, the supernatants were collected and incubated for 1 h at 56℃ with 10 mM DTT in the final concentration. The products were immediately mixed with IAM to the final concentration of 55 mM and stayed in dark for 1 h. The supernatants were collected after centrifugating at 20,000 rpm for 30 min. The protein concentrations in the final products were measured using Bradford protein assay (Sigma) and the qualities of the extracted proteins were analyzed by SDS-PAGE.

### Protein digestion and peptide purification

Aliquots of 5 mg proteins each preparation were transferred into a 10 K ultrafilter (Sartorius Stedim Lab L Ltd) and centrifuged at 4℃,4,000 rpm for 40 min. 5 ml of 5 mM Triethylammonium bicarbonate (TEBA) was added into the filters and centrifuged at 4℃, 4,000 rpm for 40 min, and this procedure was repeated two times. Protein digestion was performed with 1 μg/μl trypsin at 37℃ for 24 h. The purification of peptides was conducted with C18 reversed-phase column (Agela Technologies) to remove salts and dried by vacuum. The lengths of the majority of the digested peptides were less than 30 amino acids.

### Enrichment of the acetylated peptides

The dried products were solved in PTMScan^®^ IAP (reagents supplied in the kit) buffer containing 50 mM NaCl, 10 mM Na_3_PO_4_ and 50 mM MOPS, pH7.2. The pellets were discarded by centrifuged at 10,000 rpm at 4℃ for 5 min. By using a commercial kit (ICP0388, ImmuneChem Pharmaceuticals), the acetylated peptides were enriched and the beads were incubated with 2% acetic acid and IAP buffer overnight rotatably after washing with PBS. Finally, the beads were eluted with 0.15% TFA (reagents supplied in the kit) and peptides in supernatants were collected after centrifugating.

### Peptide TMT-labelling and identification of mass spectra

Prepared TMT-labeled reagents (Thermo Scientific) were mixed with 41 μl acetonitrile and vortexed for 1 min. Then the preparations of the tested peptide were mixed with different isotopes and incubated at RT for 1 h. The mixture was added with 8 μl 5% hydroxylamine for 15 min. Different labeled products were mixed 1:1:1:1 and dried by vacuum for further using.

Peptide mixtures were loaded onto an Acclaim PePmap C18-reversed phase column (75 μm × 2 cm, 3 μm, 100 Ǻ, Thermo Scientific) and separated with reversed phase C18 column (75 μm × 10 cm, 5 μm, 300 Ǻ, Agela Technologies) mounted on a Dionex ultimate 3 000 nano LC system. Peptides were eluted using a gradient of 5–80% (v/v) acetonitrile in 0.1% formic acid over 65 min at a flow rate of 300 nl min^−1^ combined with a Q Exactive mass spectrometer (Thermo Fisher Scientific, MA, USA).

The eluates were directly entered Q-Exactive MS (Thermo Fisher Scientific, Waltham, MA, USA), setting in positive ion mode and data-dependent manner with full MS scan from 350–2000 m/z, full scan resolution at 70,000, MS/MS scan resolution at 35,000. MS/MS scan with minimum signal threshold 1E^+5^, isolation width at 2 Da. To evaluate the performance of this mass spectrometry on the TMT labeled samples, two MS/MS acquisition modes, higher collision energy dissociation (HCD) was employed. To optimize the MS/MS acquisition efficiency of HCD, normalized collision energy (NCE) was systemically examined 30, stepped 20%.

### Bioinformatics analysis

Spectrum screening, protein identification and protein quantitative parameters used in mass spectrometry data processing were showed as following. Spectrum screening parameters included that mass range of parent ions was 350–6,000 Da, minimum number of peaks in secondary mass spectrometry was 10, signal-to-noise ratio value was 1.5. Identification retrieval parameters included that Mascot version was 2.3.0, database was uniport_2019_human 9606 (Time files compressed: Wed Jun 17 13:20:26 2020 and Number of sequences: 24,873). The quantitative parameters included that protein ratio type was median, the minimum peptide was 1 and the normalization method was median.

In this study, the peptides or the acetylated peptides in the carcinoma tissues changing > 1.2 fold compared with the data of normal control were considered as the differentially expressed peptides (DEPs) or differentially expressed acetylated peptides (DEAPs). *P* < 0.05 was generally taken as the difference screening criteria to evaluate the significance of difference in protein quantification. Volcano Plot, Gene Ontology (GO functional annotation), KEGG pathway analysis (including top 20 pathway enrichment, KEGG classification), Heat map clustering and protein interaction network were included in the further bioinformatics analysis for proteomics profiling for the global and acetylated proteins of PTC.

### Western blot

Different tumor tissues were homogenized in lysis buffer (100 mM NaCl, 10 mM EDTA, 0.5% Nonidet P-40, 0.5% sodium deoxycholate, 10 mM Tris, pH 7.5) on ice containing protease inhibitor cocktail set III (Merck Millipore, 535140-1SET). The supernatant fractions were collected after the homogenates being centrifuged at 2,000 g for 10 min, separated by 12% SDS-PAGE and electro-transferred onto nitrocellulose membranes. Membranes were incubated with various primary antibodies, including fibronectin 1 (Fib) antibody (Santa Cruz Biotechnology, sc8422), carbonic anhydrase 1 (CA1) antibody (R&D Systems, AF2180-SP), chitinase 3 like protein 1 (CHI3L1) antibody (R&D Systems, AF2599-SP) and metalloproteinase inhibitor 1 (TIMP1) antibody (Santa Cruz Biotechnology, sc365905), at 4 °C overnight and then incubated with HRP conjugated secondary antibodies (Jackson Immuno Research Labs, 115-035-003 and 111-035-003) at RT for 1 h after washing with PBS. For detecting total acetylated proteins, anti-acetylated-lysine antibody (ICP0380, Immunechem, Burnaby, BC, Canada) was used. The blots were developed using enhanced chemiluminescence (ECL) system. Images were captured by ChemiScope 6000 (CLiNX).

### Statistical analysis

The results of immunoblot images were analyzed using software Image J and gray values of target blots were evaluated. Statistical analyses were carried using Student’s t test.

## Results

### Global protein and acetylated protein profiles of tumor and normal tissues of PTC

The tissue homogenates of the surgically removed PTC from 10 patients diagnosed were pooled as the carcinoma-tumor specimen (Ca-T) and the normal thyroid tissues adjacent to the lesion from the same patients were pooled as the carcinoma-normal samples (Ca-N). The extracted acetylated proteins from tumor and normal tissues were nominated as Ca-T-A and Ca-N-A. The quality of extracted proteins, quantitative accuracy of proteins, trypsin hydrolysis efficiency, mass spectral mass deviation, mass spectrometry acquisition intensity and data volume fulfilled the requirements of the quality control for proteomic assays (Suppl Fig. 1).

**Fig. 1:**
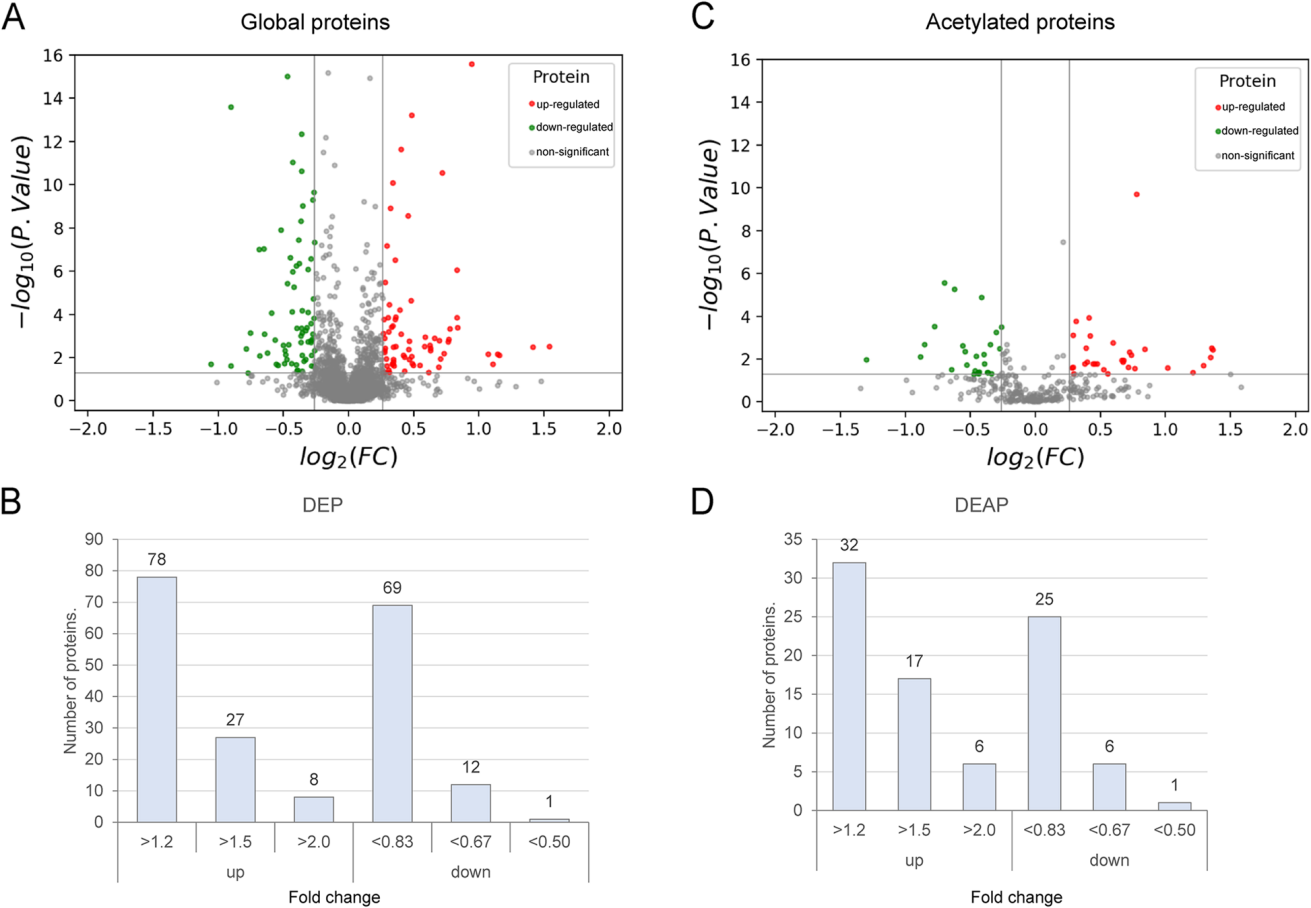
Volcano plots and numbers of DEPs identified in global proteomics and DEAPs in acetylated proteomics in comparison of Ca-T with Ca-N. Volcano plots of DEPs in global proteomics (**A**) and DEAPs in acetylated proteomics (**C**). Green dots represent the down-regulated proteins and red dots represent up-regulated ones. Gray dots are non-significantly changed proteins. The numbers of DEPs (**B**) and DEAPs (**D**). The changed folds of the up- and down-regulated DEPs and DEAPs are indicated on X-axis. The numbers of the changed proteins are indicated above the columns

For global proteomics profiling, 33,726 matched spectra were achieved, and 3,214 proteins, elicited from 11,350 unique peptides, were identified with 95% confidence interval by the Peptide Prophet Algorithm. Totally 1,923 proteins were identified both in the samples of Ca-T and Ca-N. The general pattern of DEPs and non-differential expressed proteins were showed in the Volcano plot (Fig. 1A). One hundred forty-seven proteins in Ca-T were considered as DEPs in comparison with Ca-N when utilizing the standard of 1.2-fold changed and *p* < 0.05, among them 78 DEPs were up-regulated and 69 DEPs were down-regulated, respectively. Thirty-nine DEPs showed ≥ 1.5-fold changed, including 27 up- and 12 down-regulated ones. Nine DEPs were ≥ 2.0-fold changed, including 8 up- and 1 down-regulated ones (Fig. 1B).

For acetylated proteomics profiling, 5,628 matched spectra were achieved, and 636 proteins, elicited from 1,722 unique peptides, were identified with 95% confidence interval by the Peptide Prophet Algorithm. Totally 311 proteins were identified both in the samples of Ca-N-A and Ca-T-A. The general pattern of DEAPs and non-differential ones were illustrated in the Volcano plot (Fig. 1C). Total 57 DEAPs in the sample of Ca-T-A were obtained in comparison with Ca-N-A, showing ≥ 1.2-fold changed (32 up- and 25 down-regulated ones). 23 DEAPs were 1.5-fold changed (17 up- and 6 down-regulated ones) and 7 DEAPs were ≥ 2.0-fold changed (6 up- and 1 down-regulated ones) (Fig. 1D).

**Fig. 2:**
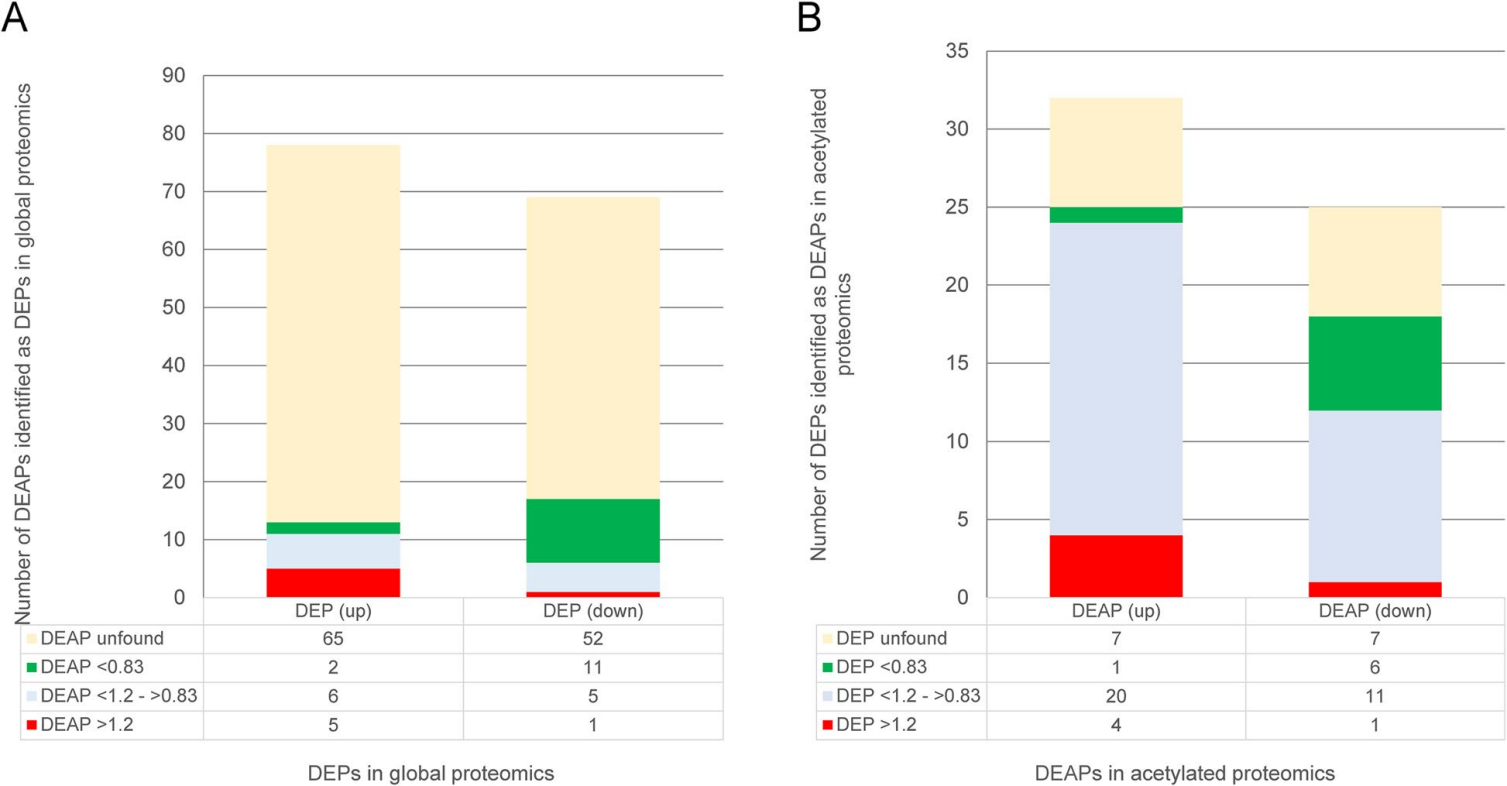
The numbers and statuses of the DEPs identifiable in acetylated proteomics (**A**) and the DEAPs identifiable in global proteomics (**B**). Yellow graphic: unfound; green graphic: downregulated < 0.83 fold; grey graphic: insignificantly changed between < 1.2 and > 0.83 fold; red graphic: upregulated > 1.2 fold. The numbers of DEPs and DEAPs identifiable in the opposite proteomic assays are separately showed on the bottom tables

In the context of global proteomics, 7.6% (147) out of 1923 identified proteins showed differentially expressed between Ca-T and Ca-N, while in the context of acetylated proteomics, 18.3% (57) out of 311 identified proteins were differentially expressed. Among 147 DEPs, only 30 proteins (20.4%) were also identifiable in acetylated proteomics, whereas 43 out of 57 DEAPs (75.4%) were also detectable in global proteomics (Fig. 2A). Among 13 up-regulated and 17 down-regulated DEPs, the up-regulated (1.2-fold), unchanged (< 1.2- to > 0.83-fold) and down-regulated (< 0.83-fold) proteins in acetylated proteomics were 5, 6, 2 and 1, 5, 11 (Fig. 2A). Among 25 up-regulated and 18 down-regulated DEAPs, the up-regulated, unchanged and down-regulated proteins in global proteomics were 4, 20, 1 and 1, 11, 6, respectively (Fig. 2B).

### Top 20 up- and down-regulated DEPs and DEAPs

The top 20 up- and down-regulated DEPs and DEAPs were summarized in Tables 1 and 2. Three of the most increased DEPs were cDNA FLJ53365 highly similar to Homo sapiens fibronectin 1, fibronectin 1 (FN1), KRT1B protein (KRT1), while three most decreased DEPs were adhesion G-protein-coupled receptor G1 (ADGRG1), cDNA FLJ53570 highly similar to Keratin, type I cytoskeletal 16 (KRT16), A-gamma globin Osilo variant, respectively (Table 1). Three up-regulated DPEs (fibronectin 1, TNC protein and epididymis secretory sperm binding protein) and five down-regulated DEPs (trefoil factor 3, hemoglobin alpha-2 globin mutant, carbonic anhydrase 1, hemoglobin subunit alpha and creatine kinase B-type) were also identifiable in acetylated proteomics. Three of the most upregulated DEAPs were ribosomal protein L18a-like protein, alpha-1-acid glycoprotein 2, eukaryotic peptide chain release factor GTP-binding subunit ERF3A, while three most downregulated ones were trefoil factor 3, thyroglobulin, histone H2B, respectively (Table 2). Among the top 20 DEAPs, 14 up- and down-regulated proteins were also detectable in global proteomics.

**Table 1:** Top 20 changed DEPs in global proteomics

	Description	DEP		DEAP	
		Ca-T vs Ca-N	*p*-value	Ca-T vs Ca-N	*p*-value
Up-regulated					
1	cDNA FLJ53365, highly similar to Homo sapiens fibronectin 1 (FN1)	6.852	0.013391	/	/
2	Fibronectin 1	3.181	8.0355E-119	1.853	3.77348E-26
3	KRT1B protein	2.912	0.002952313	/	/
4	Chitinase-3-like protein 1	2.666	0.003221145	/	/
5	Metalloproteinase inhibitor 1	2.23	0.007884891	/	/
6	Dermcidin	2.209	0.007060387	/	/
7	Thrombospondin-2	2.151	0.020179382	/	/
8	Thrombospondin type-1 domain	2.098	0.006753316	/	/
9	TNC protein	1.926	2.51100E-16	1.319	0.013279683
10	cDNA FLJ59922, highly similar to Keratin, type II cytoskeletal 5	1.784	0.000395733	/	/
11	Collagen alpha-1(XII) chain	1.779	0.000144431	/	/
12	Thrombospondin 1, isoform CRA_a	1.777	8.63908E-07	/	/
13	Ferritin	1.714	0.000459552	/	/
14	Adipocyte enhancer-binding protein 1	1.705	0.001401442	/	/
15	Ethylmalonic encephalopathy 1 isoform 3	1.699	0.001812266	/	/
16	cDNA FLJ25992 fis	1.662	0.006486008	/	/
17	Periostin isoform thy6	1.643	2.87503E-11	/	/
18	Collagen, type XII	1.631	0.011170308	/	/
19	Epididymis secretory sperm binding protein	1.627	1.42457E-29	0.84	0.24960811
20	UDP-glucose 4-epimerase	1.621	0.001662	/	/
Down-regulated					
1	Adhesion G-protein-coupled receptor G1	0.48	0.019987729	/	/
2	cDNA FLJ53570, highly similar to Keratin, type I cytoskeletal 16	0.534	0.024382628	/	/
3	A-gamma globin Osilo variant	0.535	2.444E-14	/	/
4	Huntingtin interacting protein-1	0.58	0.003860445	/	/
5	Ribosomal protein L19	0.585	0.049612805	/	/
6	cDNA, FLJ79193, highly similar to Beta-1,4-galactosyltransferase 1	0.593	0.000695376	/	/
7	Trefoil factor 3	0.621	9.53882E-08	0.406	0.010563374
8	UPF0764 protein	0.623	0.008144689	/	/
9	Hemoglobin alpha-2 globin mutant	0.638	8.85393E-08	0.728	0.268025154
10	Sulfotransferase	0.639	0.000824192	/	/
11	60S ribosomal protein L6	0.65	0.006101454	/	/
12	Sulfhydryl oxidase	0.663	8.65178E-05	/	/
13	Carbonic anhydrase 1	0.67	5.56952E-27	0.65	5.44636E-06
14	Retinoid-inducible serine carboxypeptidase	0.676	0.00147738	/	/
15	ER membrane protein complex subunit 1	0.68	0.019411598	/	/
16	Erythrocyte membrane protein band 4.2	0.687	0.022202241	/	/
17	Hemoglobin subunit alpha	0.692	1.60939E-42	0.694	0.019044979
18	Creatine kinase B-type	0.697	1.23955E-08	0.805	0.17691894
19	cDNA FLJ51076, weakly similar to LIM domain only protein 7	0.707	0.002702647	/	/
20	cDNA FLJ76704, highly similar to Homo sapiens leukemia inhibitory factor receptor (LIFR)	0.71	0.018325546	/	/

**Table 2:** Top 20 changed DEAPs in acetylated proteomics

	Description	DEAP		DEP	
		Ca-T vs Ca-N	*p*-value	Ca-T vs Ca-N	*p*-value
Up-regulated					
1	Ribosomal protein L18a-like protein	2.572	0.003707381	/	/
2	Alpha-1-acid glycoprotein 2	2.557	0.00306434	1.19	0.012107743
3	Eukaryotic peptide chain release factor GTP-binding subunit ERF3A	2.538	0.008127735	1.006	0.525148945
4	Glyoxalase domain-containing protein 4	2.449	0.020169829	/	/
5	Aminopeptidase B	2.314	0.043350089	1.014	0.884044368
6	Importin-7	2.025	0.025222666	/	/
7	Fibronectin 1	1.853	3.77348E-26	3.181	8.0355E-119
8	Calreticulin variant	1.789	0.003447804	0.936	5.44652E-06
9	Phosphatidylethanolamine-binding protein 1	1.714	1.89684E-10	0.939	0.125084426
10	Moesin	1.695	0.027378163	1.091	1.27729E-05
11	Vesicular integral-membrane protein VIP36	1.665	0.006603029	0.93	0.049104234
12	Periostin, osteoblast specific factor, isoform CRA_a	1.652	0.004624051	/	/
13	Profilin	1.638	0.02402358	1.034	0.091470985
14	Reticulon 4 receptor-like 2	1.597	0.0110701	0.756	5.62348E-07
15	GTP-binding nuclear protein Ran	1.595	0.013634802	1.012	0.40861548
16	Glucose-6-phosphate isomerase	1.583	0.011011616	0.905	1.40188E-05
17	Glutathione S-transferase	1.511	0.001672493	/	/
18	Dipeptidyl peptidase 2	1.469	0.046930984	1.085	0.323364607
19	ADP-ribosylation factor 5	1.439	0.031271204	/	/
20	ADP-ribosylation factor 4	1.387	0.016834738	0.945	0.482804157
Down-regulated					
1	Trefoil factor 3	0.406	0.010563374	0.621	9.539E-08
2	Thyroglobulin	0.542	0.007670257	0.993	0.630028771
3	Histone H2B	0.554	0.002104	0.917	0.000105677
4	Uncharacterized protein DKFZp686G03277	0.583	0.000299479	/	/
5	Histone H2B type 1-O	0.615	2.73965E-06	/	/
6	Small nuclear ribonucleoprotein Sm D2	0.64	0.0300279	0.901	0.212725423
7	Carbonic anhydrase 1	0.65	5.44636E-06	0.67	5.56952E-27
8	cDNA FLJ46620 fis, clone TLUNG2000654, highly similar to Keratin, type II cytoskeletal 7	0.679	0.002421842	/	/
9	Liver histone H1e	0.686	0.00438445	/	/
10	Hemoglobin subunit alpha	0.694	0.019044979	0.692	1.60939E-42
11	Collagen alpha-1(VI) chain	0.72	0.04994192	0.919	0.004203776
12	Plectin	0.724	0.034564102	1.118	1.17189E-15
13	Heterogeneous nuclear ribonucleoprotein A0	0.729	0.00721017	/	/
14	Phosphoglycerate kinase	0.735	0.04306316	0.927	1.23498E-11
15	40S ribosomal protein S2	0.737	0.046694313	1.028	0.361381311
16	Phosphoglucomutase-2	0.74	0.038894305	0.969	0.171058694
17	Collagen alpha-2(I) chain	0.749	1.327E-05	1.263	8.25091E-11
18	Fatty acid-binding protein	0.759	0.006083165	0.855	0.012262904
19	cDNA FLJ52561, highly similar to Four and a half LIM domains protein 1	0.761	0.016453591	/	/
20	Immunoglobulin heavy constant gamma 3	0.774	0.041659375	0.97	0.013691918

*/* Not identified

### Hierarchical clustering heat map

**Fig. 3:**
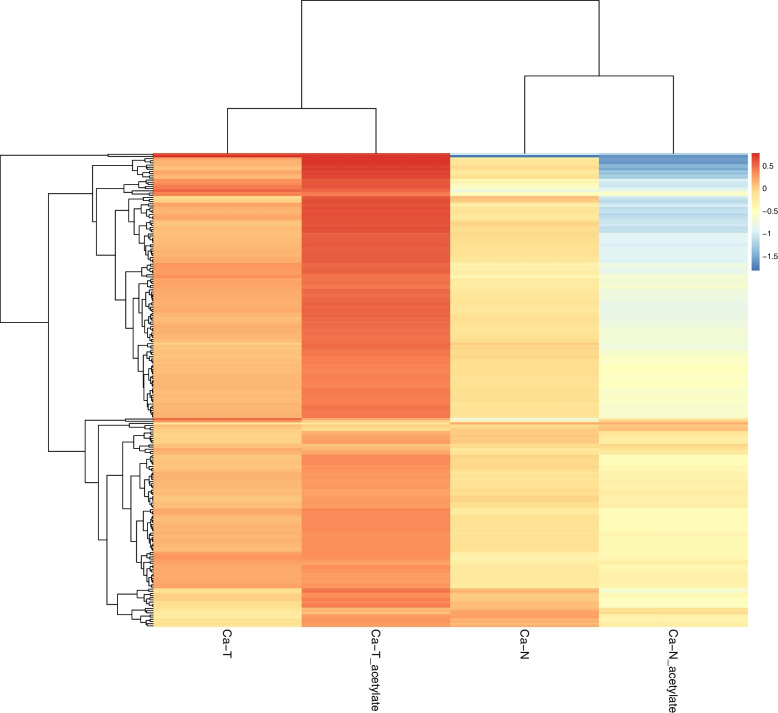
Hierarchical clustering analysis for the proteins identified in global and acetylated proteomics

Totally 221 proteins from Ca-T and Ca-N samples were identified both in global and acetylated proteomics. To see the general profile of those proteins in global and acetylated proteomics between the groups of Ca-T and Ca-N, hierarchical clustering was performed. As shown in Fig. 3, the profile of acetylated proteomics between the groups of Ca-T and Ca-N revealed a more pronounced change than that of global proteomics. More proteins displayed increased acetylated profiling in Ca-T and decreased acetylated profiling in Ca-N.

### Validation of the selected DEPs and DEAPs by Western blots

**Fig. 4:**
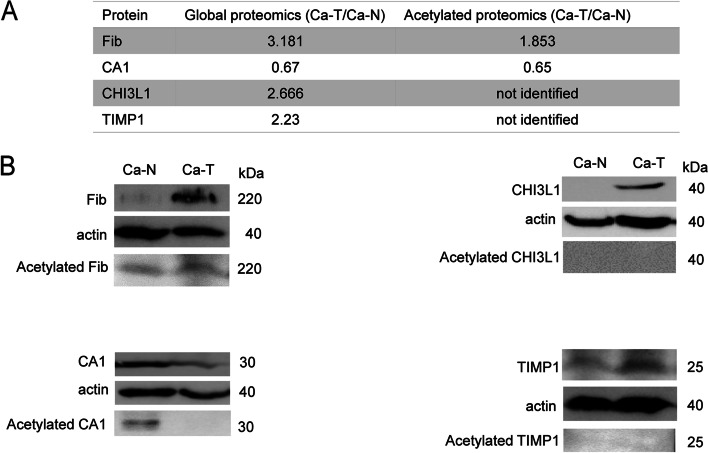
Western blot validation of four selected proteins (Fib, CA1, CHI3L1, TIMP1) identified in omic assays. **A** The changed folds or statuses of the four selected proteins in omics assays. **B** Western blots. Upper blots each panel: the extracts from Ca-T and Ca-N samples blotted with the specific antibodies against individual target proteins. Middle blots each panel: the extracts from Ca-T and Ca-N samples blotted with the antibody to β-actin. Lower blots each panel: the acetylated extracts from Ca-T and Ca-N samples blotted with the specific antibodies against individual target proteins

To validate the screening results for DEPs and DEAPs based on MS/MS, the expressions of four proteins, including fibronectin 1 (Fib), carbonic anhydrase 1 (CA1), chitinase 3 like protein 1 (CHI3L1) and metalloproteinase inhibitor 1 (TIMP1), in the samples of Ca-T and Ca-N were evaluated by the Western blots. The changed states of those four proteins of Ca-T vs Ca-N in global proteomics and acetylated proteomics were 3.181 and 1.853 (Fib), 0.67 and 0.65 (CA1), 2.666 and undetectable (CHI3L1), 2.23 and undetectable (TIMP1), respectively (Fig. 4A). Western blots revealed that the signals of Fib, CHI3L1 and TIMP1 in the samples of Ca-T were stronger than that of Ca-N, while the band of CA1 in Ca-T sample was much weaker (Fig. 4B). Further, the acetylated extracts from Ca-T and Ca-N were prepared and subjected into the Western blots with individual specific antibodies. As shown in Fig. 4B, the signal of acetylated Fib in Ca-T was stronger than that of Ca-N. Acetylated CA1 band was observed only in Ca-N. No specific acetylated CHI3L1 and TIMP1 signal was identified both in Ca-T and Ca-N tissues. Those data validate, at least partially, the results of the proteomic assays.

### Involvement of the significant pathways

To analyze the involved metabolic and signaling pathways by DEPs or DEAPs in tumor tissues, Kyoto Encycloppedia of Genes and Genomes (KEGG) pathway analyses were conducted using software of KOBAS2.0. In global proteomics, 78 pathways were involved but only 4 pathways were significantly changed (*P* < 0.05), including mineral absorption, protein digestion and absorption, nicotinate and nicotinamide metabolism and relax in signaling pathway (Table 3). In acetylated proteomics, 37 pathways were identified, among them 3 pathways showed significance (*P* < 0.05), including proteoglycans in cancer, viral carcinogenesis and tight junction (Table 3).

**Table 3:** KEGG pathways based on DEPs and DEAPs

	Pathway	*P*-value	Protein in Diff Exp		Protein in Background
Global proteomics					
1	Mineral absorption	0.000967	3	Cytochrome b reductase 1 (0.726), Metallothionein-2 (0.715), Ferritin light chain (1.612)	5
2	Protein digestion and absorption	0.009736	3	Collagen alpha-1(III) chain (1.258), Collagen alpha-1(I) chain (1.247), Dipeptidyl peptidase 4 (1.547)	10
3	Nicotinate and nicotinamide metabolism	0.020222	2	Purine nucleoside phosphorylase (1.27), Nicotinamide N-methyltransferase (1.402)	5
4	Relaxin signaling pathway	0.036907	3	Collagen alpha-1(III) chain (1.258), Collagen alpha-1(I) chain (1.247), Guanine nucleotide-binding protein G(I)/G(S)/G(O) subunit gamma-12 (0.761)	16
Acetylated proteomics					
1	Proteoglycans in cancer	0.002704	3	Decorin (1.299), Lumican (1.22), Moesin (1.695)	4
2	Viral carcinogenesis	0.011823	3	Histone H2B type 1-O (0.615), Complement C3 (1.221), 14-3-3 protein theta (1.215)	6
3	Tight junction	0.04352	2	Moesin (1.695), Myosin regulatory light chain 12A (0.81)	4

### GO ontology description

To characterize the distributions of DEPs and DEAPs in cellular processes, GO analysis was conducted and the significantly affected processes (*P* < 0.05) were counted. In the categories of cellular component, biological process and molecular function, the numbers of the affected processes based on global proteomics were 67, 400 and 73, while those based on acetylated proteomics were 28, 121 and 3, respectively.

**Fig. 5:**
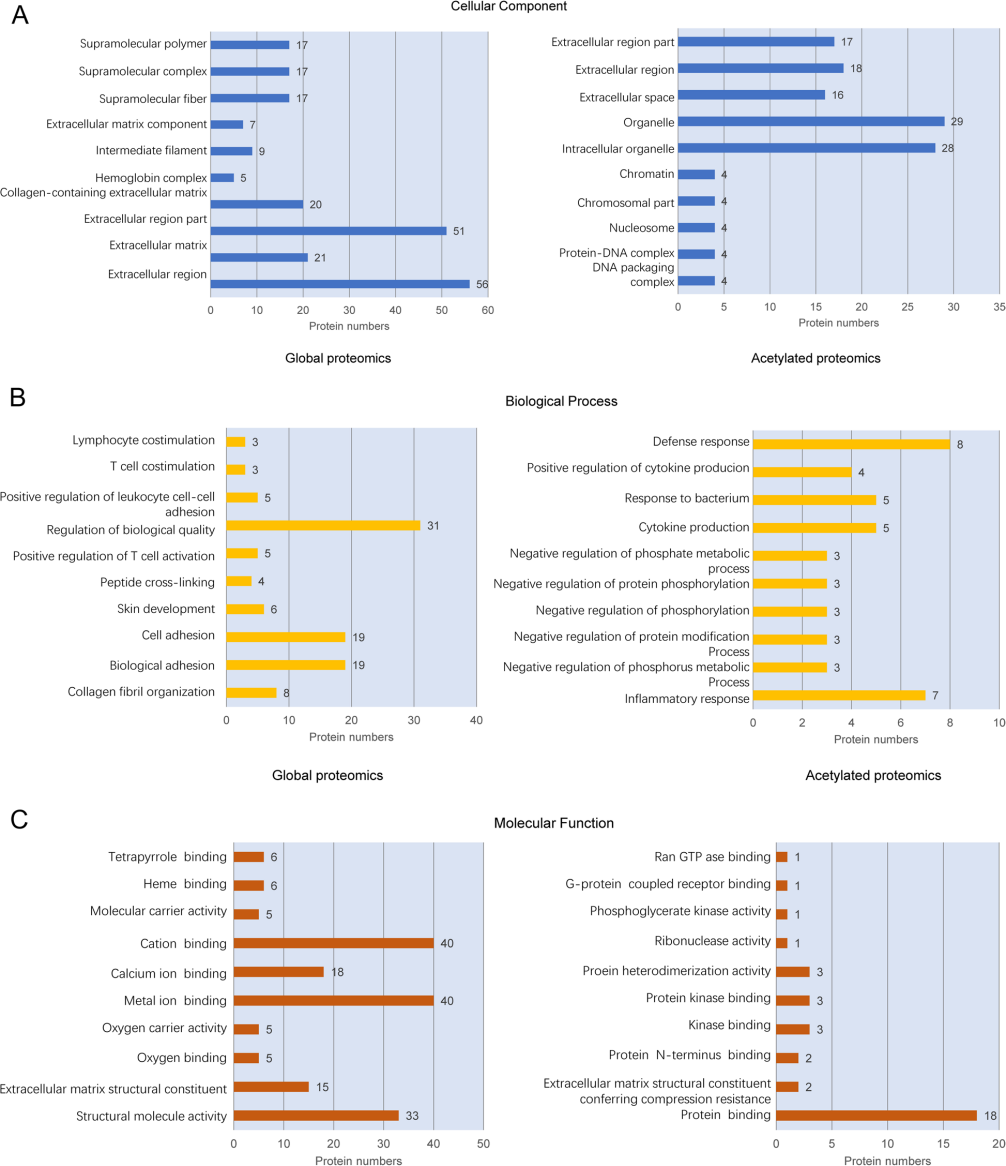
Top 10 affected processes in GO analysis based on the DEPs and DEAPs in Ca-T compared to Ca-N samples. **A** Category of cellular component. **B** Category of biological process. **C** Category of molecular function. The numbers of the significantly changed proteins each process are indicated on the columns

The top ten affected biological processes each category based on the *P* values and the involved protein numbers in global and acetylated proteomics were separately calculated. In the category of cellular component (Fig. 5A), they were extracellular region, extracellular matrix, extracellular region part, collagen-containing extracellular matrix, hemoglobin complex, intermediate filament, extracellular matrix component, supramolecular fiber, supramolecular complex and supramolecular polymer in global proteomics, while DNA packaging complex, protein-DNA complex, nucleosome, chromosomal part, chromatin, intracellular organelle, organelle, extracellular space, extracellular region and extracellular region part in acetylated proteomics. Two processes (extracellular region and extracellular region part) were identified in both global and acetylated proteomics.

In the category of biological process (Fig. 5B), the top ten affected functions were collagen fibril organization, biological adhesion, cell adhesion, skin development, peptide cross-linking, positive regulation of T cell activation, regulation of biological quality, positive regulation of leukocyte cell-cell adhesion, T cell co-stimulation and lymphocyte co-stimulation in global proteomics, while inflammatory response, negative regulation of phosphorus metabolic process, negative regulation of protein modification process, negative regulation of phosphorylation, negative regulation of protein phosphorylation, negative regulation of phosphate metabolic process, cytokine production, response to bacterium, positive regulation of cytokine production and defense response in acetylated proteomics. These top 10 processes were not overlap between two omics assays.

In the category of molecular function (Fig. 5C), the top ten affected functions were structural molecule activity, extracellular matrix structural constituent, oxygen binding, oxygen carrier activity, metal ion binding, calcium ion binding, cation binding, molecular carrier activity, heme binding, tetrapyrrole binding in global proteomics, while protein binding, extracellular matrix structural constituent conferring compression resistance, protein N-terminus binding, kinase binding, protein kinase binding, protein heterodimerization activity, ribonuclease activity, phosphoglycerate kinase activity, G-protein coupled receptor binding and Ran GTPase binding in acetylated proteomics. None of the top 10 processes overlapped in two omics assays.

## Discussion

In this study the global expressing proteomic and acetylated proteomic profiles of the tumor tissues of PTC were analyzed compared to the normal tissues adjacent to the lesions. Although the data in this study are not obtained from the proteomic assay of individual cases, pooled samples from 10 cases of PTC with the same TNM stage (III) might, to some extents, balance the individual differences. More proteins and differentially expressed ones has been identified in the assay of global proteomics than that of acetylated proteomics. We believe that it is reasonable, as compared with thousands of proteins there are about 1,750 proteins acetylated nearly at 3,600 sites [13, 14]. Another possibility may drive from the preparing processes for mass spectrometry, as the enrichment process of the acetylated peptides from the tested samples is conducted after the extraction of the whole proteins, which may result in loss of some peptides. On the other hand, we have found that 75.4% of DEAPs are detectable in global proteomics, while only 20.4% DEPs are seen in acetylated proteomics. It highlights that the extraction of acetylated peptides may help to enrich those of relatively low abundance.

Obviously, there is a small portion of the differentially expressed proteins can be identified both in global and acetylated proteomics. 7 out of 40 mostly changed DEPs are detectable in acetylated proteomics and 6 of them are considered as DEAPs. 28 out of 40 mostly changed DEAPs are identifiable in global proteomics and only 6 are classified as DEPs. Only two DEAPs show different altering direction, i.e., reticulon 4 receptor-like 2 (1.579 in acetylated and 0.756 in global proteomics) and collagen alpha-2(I) chain (0.749 in acetylated and 1.263 in global proteomics). The rest of the mostly changed DEPs and DEAPs shows the same changing direction, or maintains unchanged, or is undetectable in the opposite technique.

We have also reviewed the top 10 up- and down-regulated DEPs and DEAPs in thyroid cancer and other cancers from literatures and summarized the results in Table 4. All 10 of the mostly up-regulated DEPs in this study have already addressed in other types of cancers showing overexpression, i.e., fibronectin 1 [7, 15], chitinase-3-like protein 1 [16, 17], metalloproteinase inhibitor 1 [18, 19], thrombospondin-2 [20, 21], thrombospondin type 1 [22, 23], TNC protein [24, 25], KRT1B protein, dermcidin [26], keratin, type II cytoskeletal 5 [27]. Among them, KRT1B protein, dermcidin and keratin, type II cytoskeletal 5 are not well addressed in thyroid cancer. Except UPF0764 protein and A-gamma globin Osilo variant, 8 of the top 10 down-regulated DEPs have also mentioned in other types of cancers, adhesion G-protein-coupled receptor G1 (decreased) [28], keratin-type I cytoskeletal 16 [27], Huntingtin interacting protein-1 (decreased) [29], ribosomal protein L19 (increased) [30], Beta-1,4-galactosyltransferase 1 (negatively regulates cell survival) [31], trefoil factor 3 (decreased) [32, 33], hemoglobin alpha-2 globin mutant [34], sulfotransferase [35, 36]. Contrarily, only 2 proteins, trefoil factor 3 and sulfotransferase are addressed in thyroid cancer.

**Table 4:** The top 10 up- and down-regulated DEPs and DEAPs addressed in the literatures

Proteomics	Change	Protein	Papillary thyroid carcinoma	Other cancers
Global	Up-regulated	Fibronectin 1	Y, increased [1]	Y, increased [2]
		cDNA FLJ53365, highly similar to fibronectin 1, transcript variant 4	Y, increased	Y, increased
		KRT1B protein	N	Y, increased [3]
		Chitinase-3-like protein 1	Y, increased [4]	Y, increased [5]
		Metalloproteinase inhibitor 1	Y, increased [6]	Y, increased [7]
		Dermcidin	N	Y, increased [8]
		Thrombospondin-2	Y, increased [9]	Y, increased [8]
		Thrombospondin type 1	Y, increased [10]	Y, increased [11]
		TNC protein	Y, increased [12]	Y, increased [13]
		Keratin, type II cytoskeletal 5	N	Y, increased [14]
	Down-regulated	Adhesion G-protein-coupled receptor G1	N	Y, [15]
		Keratin, type I cytoskeletal 16	N	Y, [14]
		A-gamma globin Osilo variant	N	N
		Huntingtin interacting protein-1	N	Y, [16]
		Ribosomal protein L19	N	Y, [17]
		Beta-1,4-galactosyltransferase 1	N	Y, [18]
		Trefoil factor 3	Y, decreased [19]	Y, decreased [20]
		UPF0764 protein	N	N
		Hemoglobin alpha-2 globin mutant	N	Y, [21]
		Sulfotransferase	Y, [22]	Y, [23]
Acetylated	Up-regulated	Ribosomal protein L18a-like protein	N	N
		Alpha-1-acid glycoprotein 2	N	Y, [24]
		Eukaryotic peptide chain release factor GTP-binding subunit ERF3A	N	N
		Glyoxalase domain-containing protein 4	N	N
		Aminopeptidase B	N	N
		Importin-7	N	N
		Fibronectin 1	N	N
		Calreticulin variant	N	N
		Phosphatidylethanolamine-binding protein 1	N	N
		Moesin	N	N
	Down-regulated	Trefoil factor 3	N	N
		Thyroglobulin	N	N
		Histone H2B	N	Y, [25]
		Uncharacterized protein DKFZp686G03277	N	N
		Histone H2B type 1-O	N	N
		Small nuclear ribonucleoprotein Sm D2	N	N
		Carbonic anhydrase 1	N	N
		Keratin, type II cytoskeletal 7	N	N
		Liver histone H1e	N	N
		Hemoglobin subunit alpha	N	N

Unlike the proteomic assays for cancers, the protein acetylation status in carcinomas is relatively poorly understood. Reviewing the top 10 increased and decreased DEAPs in this study from the literatures (Table 4), we find a few of proteins having their acetylated data, such as alpha-1-acid glycoprotein 2 in cystic ovarian tumors [37] and histone H2B in a bioluminescent orthotopic surgical xenograft model of ovarian cancer [38], although majority of them has been proposed to be associated with various malignant tumors. Notably, three acetylated forms of histone proteins, H2B, H2B type 1-O and liver H1e, are down-regulated in the tumor tissues. Acetylation and deacetylation of histone are the two important processes amongst the different modes of epigenetic modulation that are involved in regulating cancer initiation and development [39]. Abnormal expression of histone deacetylases (HDACs) is often reported in various types of cancers, e.g., overexpression of HDAC1-3 in ovarian cancer, overexpression of HDAC 1 and 3 in lung cancer and overexpression of HDAC2 in gastric cancer [39]. Overexpression of HDAC leads to enhanced deacetylation of histones, and subsequently increases the gravitational attraction between DNA and histones and compacts the loose nucleosome against the expression of certain genes, including some tumor suppressor genes, which makes HDAC inhibitors be potent drug molecules [40]. Low acetylated levels of different histones in tumor tissues in comparison with the adjacent normal tissues in this study may also reflect an active status of HDACs in papillary thyroid cancer, though the level of HDAC in tumor tissues seems not change significantly in comparison with that of normal tissues by global proteomics (Ca-T/ Ca-N, 0.937 in histone deacetylase and 1.065 in histone deacetylase complex subunit SAP18, respectively).

Functional GO annotation analysis and KEGG pathway analysis based on the DEPs and DEAPs show completely different pictures, in which only two biological processes are commonly seen among the top 10 involved processes in the context of three categories of GO analysis. We believe that it is because of the difference in the proteins differentially expressed in the assays of global and acetylated proteomics. Global proteomics evaluates the differences in the components and alterations of proteins among the tested samples, whereas acetylated proteomics reflects the difference in protein acetylation, one of the protein post-translational processes,. Taken the whole profiling of those two proteomics together will give us more broad view of protein alterations on the carcinogenesis of PTC.

## Conclusions

In this study, we have, for the first time, screened the profiles of global and acetylated proteomics of PTC controlled with the normal tissues adjacent to the lesions in parallel. In total, 147 proteins in global proteomics and 57 proteins in acetylated proteomics in tumor tissues are considered as differentially expressed. Only small proportion of the significantly changed proteins are found both in global and acetylated proteomics, leading to only two out of the top 10 processes overlap in GO assay and no pathway overlap in KEGG pathway assay. As global proteomics evaluates the changes of protein levels and acetylated proteomics reflects the alterations of protein post-translation affecting protein activity, evaluation of the changes in those two proteomics techniques together will help to reveal the pathogenesis of PTC more comprehensively.

## Supplementary Information

**Additional file 1: Supplement Figure 1.** Protein quality control. A. Protein quantitative standard curve. B. Protein SDS-PAGE gel map. C. Peptide length distribution diagram.

**Additional file 2: Supplemental Table 1.** The ages and TNM stages of 10 enrolled patients of PTC

## Data Availability

The data and material are available.

## Abbreviations

Ac-CoA: Acetyl-coenzyme A
Ca-T: Sample of the tumor tissue of PTC
Ca-N: Sample of the adjacent normal tissues of PTC
DEAPs: Differentially expressed acetylated peptides
DEPs: Differentially expressed proteins
GO: Gene ontology
KEGG: Kyoto Encycloppedia of Genes and Genomes
PTC: Papillary thyroid carcinoma
PTMs: Post-translational modifications

## References

[1] Dralle H, Machens A, Basa J, Fatourechi V, Franceschi S, Hay ID (2015). Follicular cell-derived thyroid cancer. Nat Rev Dis Primers.

[2] Lin JS, Bowles EJA, Williams SB, Morrison CC (2017). Screening for thyroid cancer: updated evidence report and systematic review for the US Preventive Services Task Force. JAMA.

[3] Mattiuzzi C, Lippi G (2019). Current cancer epidemiology. J Epidemiol Glob Health.

[4] Wiltshire JJ, Drake TM, Uttley L, Balasubramanian SP (2016). Systematic review of trends in the incidence rates of thyroid cancer. Thyroid.

[5] Kim J, Gosnell JE, Roman SA (2020). Geographic influences in the global rise of thyroid cancer. Nat Rev Endocrinol.

[6] Kalfert D, Ludvikova M, Kholova I, Ludvik J, Topolocan O, Plzak J (2020). Combined use of galectin-3 and thyroid peroxidase improves the differential diagnosis of thyroid tumors. Neoplasma.

[7] Sponziello M, Rosignolo F, Celano M, Maggisano V, Pecce V, De Rose RF (2016). Fibronectin-1 expression is increased in aggressive thyroid cancer and favors the migration and invasion of cancer cells. Mol Cell Endocrinol.

[8] Liu YY, Morreau H, Kievit J, Romijn JA, Carrasco N, Smit JW (2008). Combined immunostaining with galectin-3, fibronectin-1, CITED-1, Hector Battifora mesothelial-1, cytokeratin-19, peroxisome proliferator-activated receptor-{gamma}, and sodium/iodide symporter antibodies for the differential diagnosis of non-medullary thyroid carcinoma. Eur J Endocrinol.

[9] Prasad ML, Pellegata NS, Huang Y, Nagaraja HN, de la Chapelle A, Kloos RT (2005). Galectin-3, fibronectin-1, CITED-1, HBME1 and cytokeratin-19 immunohistochemistry is useful for the differential diagnosis of thyroid tumors. Mod Pathol.

[10] Tardiolo G, Bramanti P, Mazzon E (2018). Overview on the effects of N-acetylcysteine in neurodegenerative diseases. Molecules.

[11] Li D, Yang Y, Wang S, He X, Liu M, Bai B (2021). Role of acetylation in doxorubicin-induced cardiotoxicity. Redox Biol.

[12] Gil J, Ramirez-Torres A, Encarnacion-Guevara S (2017). Lysine acetylation and cancer: a proteomics perspective. J Proteomics.

[13] Deng W, Wang C, Zhang Y, Xu Y, Zhang S, Liu Z (2016). GPS-PAIL: prediction of lysine acetyltransferase-specific modification sites from protein sequences. Sci Rep.

[14] Carrico C, Meyer JG, He W, Gibson BW, Verdin E (2018). The mitochondrial acylome emerges: proteomics, regulation by sirtuins, and metabolic and disease implications. Cell Metab.

[15] Zhou Y, Cao G, Cai H, Huang H, Zhu X (2020). The effect and clinical significance of FN1 expression on biological functions of gastric cancer cells. Cell Mol Biol (Noisy-le-grand).

[16] Cheng SP, Lee JJ, Chang YC, Lin CH, Li YS, Liu CL (2020). Overexpression of chitinase-3-like protein 1 is associated with structural recurrence in patients with differentiated thyroid cancer. J Pathol.

[17] Salomon J, Piotrowska A, Matusiak L, Dziegiel P, Szepietowski JC (2018). Chitinase-3-like protein 1 (YKL-40) expression in squamous cell skin cancer. Anticancer Res.

[18] Lorenc Z, Waniczek D, Lorenc-Podgorska K, Krawczyk W, Domagala M, Majewski M (2017). Profile of Expression of Genes Encoding Matrix Metallopeptidase 9 (MMP9), Matrix Metallopeptidase 28 (MMP28) and TIMP Metallopeptidase Inhibitor 1 (TIMP1) in Colorectal Cancer: Assessment of the Role in Diagnosis and Prognostication. Med Sci Monit.

[19] Bumber B, Marjanovic Kavanagh M, Jakovcevic A, Sincic N, Prstacic R, Prgomet D (2020). Role of matrix metalloproteinases and their inhibitors in the development of cervical metastases in papillary thyroid cancer. Clin Otolaryngol.

[20] Byrling J, Kristl T, Hu D, Pla I, Sanchez A, Sasor A (2020). Mass spectrometry-based analysis of formalin-fixed, paraffin-embedded distal cholangiocarcinoma identifies stromal thrombospondin-2 as a potential prognostic marker. J Transl Med.

[21] de Fraipont F, Nicholson AC, Feige JJ, Van Meir EG (2001). Thrombospondins and tumor angiogenesis. Trends Mol Med.

[22] Sid B, Langlois B, Sartelet H, Bellon G, Dedieu S, Martiny L (2008). Thrombospondin-1 enhances human thyroid carcinoma cell invasion through urokinase activity. Int J Biochem Cell Biol.

[23] Yee KO, Connolly CM, Duquette M, Kazerounian S, Washington R, Lawler J (2009). The effect of thrombospondin-1 on breast cancer metastasis. Breast Cancer Res Treat.

[24] Gocheva V, Naba A, Bhutkar A, Guardia T, Miller KM, Li CM (2017). Quantitative proteomics identify Tenascin-C as a promoter of lung cancer progression and contributor to a signature prognostic of patient survival. Proc Natl Acad Sci USA.

[25] Tseleni-Balafouta S, Gakiopoulou H, Fanourakis G, Voutsinas G, Balafoutas D, Patsouris E (2006). Tenascin-C protein expression and mRNA splice variants in thyroid carcinoma. Exp Mol Pathol.

[26] Stewart GD, Skipworth RJ, Ross JA, Fearon K, Baracos VE (2008). The dermcidin gene in cancer: role in cachexia, carcinogenesis and tumour cell survival. Curr Opin Clin Nutr Metab Care.

[27] Schumann H, Roth W, Has C, Volz A, Erfurt-Berge C, Magin TM (2012). Verrucous carcinoma in epidermolysis bullosa simplex is possibly associated with a novel mutation in the keratin 5 gene. Br J Dermatol.

[28] Yang L, Xu L (2012). GPR56 in cancer progression: current status and future perspective. Future Oncol.

[29] Hsu CY, Lin CH, Jan YH, Su CY, Yao YC, Cheng HC (2016). Huntingtin-interacting protein-1 is an early-stage prognostic biomarker of lung adenocarcinoma and suppresses metastasis via Akt-mediated epithelial-mesenchymal transition. Am J Respir Crit Care Med.

[30] Bee A, Ke Y, Forootan S, Lin K, Beesley C, Forrest SE (2006). Ribosomal protein l19 is a prognostic marker for human prostate cancer. Clin Cancer Res.

[31] Tang W, Weng S, Zhang S, Wu W, Dong L, Shen X (2013). Direct interaction between surface beta1,4-galactosyltransferase 1 and epidermal growth factor receptor (EGFR) inhibits EGFR activation in hepatocellular carcinoma. Biochem Biophys Res Commun.

[32] Abols A, Ducena K, Andrejeva D, Sadovska L, Zandberga E, Vilmanis J (2015). Trefoil factor 3 is required for differentiation of thyroid follicular cells and acts as a context-dependent tumor suppressor. Neoplasma.

[33] Espinoza I, Agarwal S, Reddy A, Shenoy V, Subramony C, Sakiyama M (2021). Expression of trefoil factor 3 is decreased in colorectal cancer. Oncol Rep.

[34] Li X, Wu Z, Wang Y, Mei Q, Fu X, Han W (2013). Characterization of adult alpha- and beta-globin elevated by hydrogen peroxide in cervical cancer cells that play a cytoprotective role against oxidative insults. PLoS One.

[35] Kalathas D, Theocharis DA, Bounias D, Kyriakopoulou D, Papageorgakopoulou N, Stavropoulos MS (2009). Alterations of glycosaminoglycan disaccharide content and composition in colorectal cancer: structural and expressional studies. Oncol Rep.

[36] Schulten HJ, Al-Mansouri Z, Baghallab I, Bagatian N, Subhi O, Karim S (2015). Comparison of microarray expression profiles between follicular variant of papillary thyroid carcinomas and follicular adenomas of the thyroid. BMC Genomics.

[37] Kolwijck E, Engelke UF, van der Graaf M, Heerschap A, Blom HJ, Hadfoune M (2009). N-acetyl resonances in in vivo and in vitro NMR spectroscopy of cystic ovarian tumors. NMR Biomed.

[38] Helland O, Popa M, Bischof K, Gjertsen BT, McCormack E, Bjorge L (2016). The HDACi Panobinostat shows growth inhibition both in vitro and in a bioluminescent orthotopic surgical xenograft model of ovarian cancer. PLoS One.

[39] Shetty MG, Pai P, Deaver RE, Satyamoorthy K, Babitha KS (2021). Histone deacetylase 2 selective inhibitors: a versatile therapeutic strategy as next generation drug target in cancer therapy. Pharmacol Res.

[40] Ramaiah MJ, Tangutur AD, Manyam RR (2021). Epigenetic modulation and understanding of HDAC inhibitors in cancer therapy. Life Sci.

