# Supplementary material for: Proteomics profiling for the global and acetylated proteins of papillary thyroid cancers
Source: Proteome Sci. 2023 Apr 26;21:6. doi: 10.1186/s12953-023-00207-8 (PMC10131382; doi:10.1186/s12953-023-00207-8)
Supplement: Supplementary file 1 — Additional file 1: Supplement Figure 1. Protein quality control. A. Protein quantitative standard curve. B. Protein SDS-PAGE gel map. C. Peptide length distribution diagram. [file 12953_2023_207_MOESM1_ESM.pptx]

## Slide 1
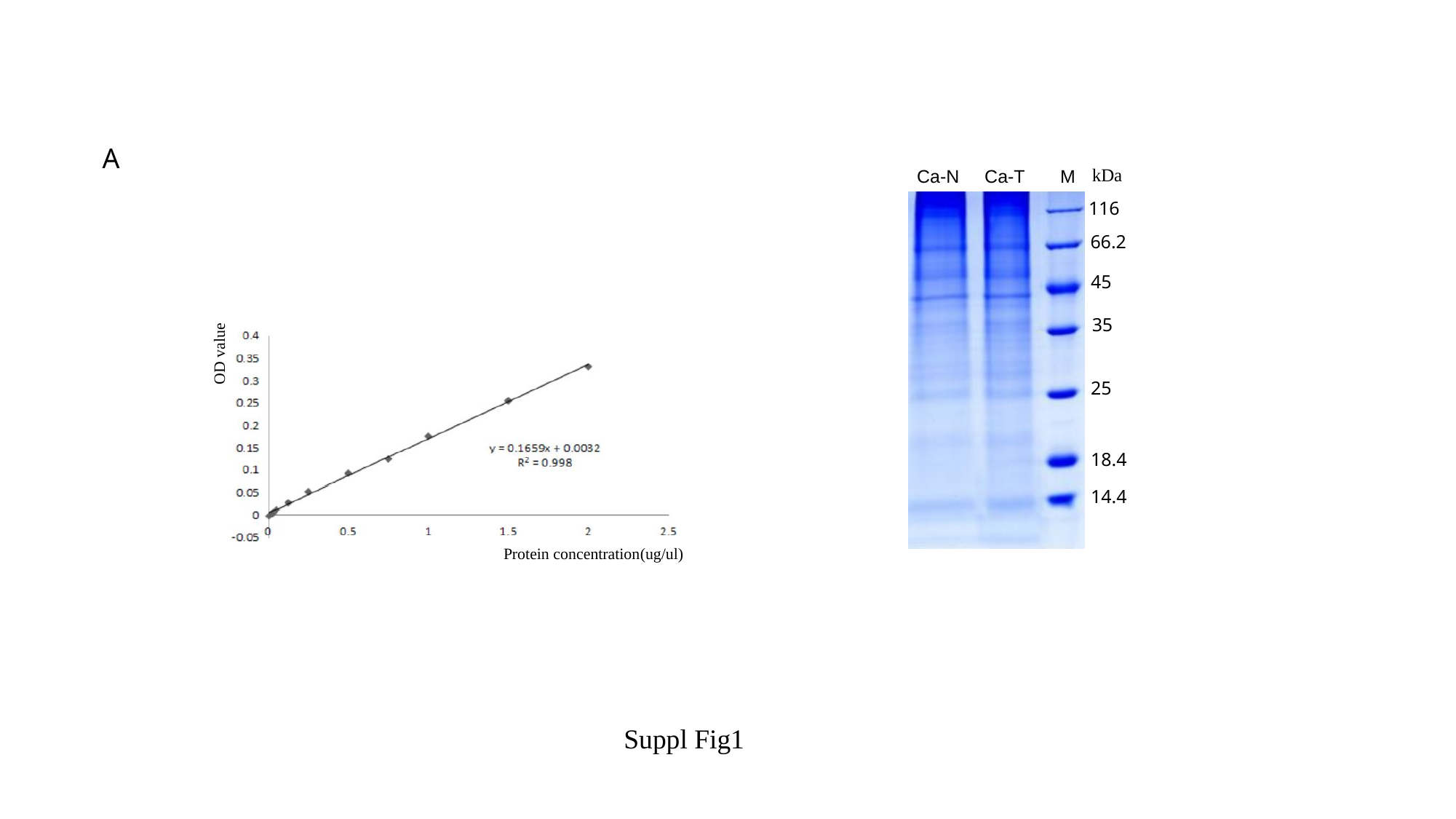

A
kDa
Ca-N Ca-T M
116
66.2
45
OD value
35
25
18.4
14.4
Protein concentration(ug/ul)
Suppl Fig1

## Slide 2
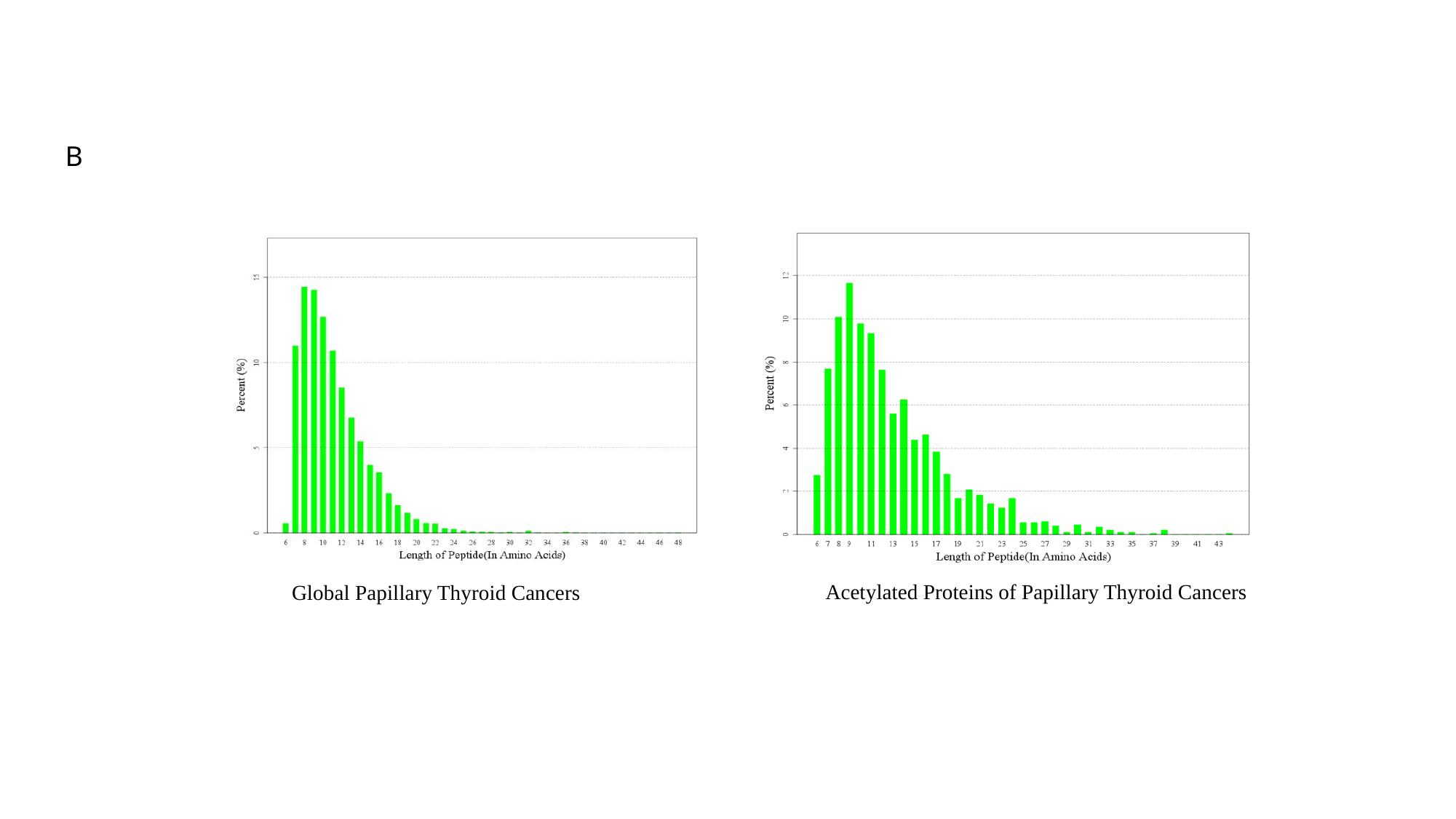

B
Acetylated Proteins of Papillary Thyroid Cancers
Global Papillary Thyroid Cancers
