# Supplementary material for: Proteomics profiling for the global and acetylated proteins of papillary thyroid cancers
Source: Proteome Sci. 2023 Apr 26;21:6. doi: 10.1186/s12953-023-00207-8 (PMC10131382; doi:10.1186/s12953-023-00207-8)
Supplement: Supplementary file 2 — Additional file 2: Supplemental Table 1. The ages and TNM stages of 10 enrolled patients of PTC [file 12953_2023_207_MOESM2_ESM.docx]

Supplemental Table 1. The ages and TNM stages of 10 enrolled patients of PTC

|  | Age (year-old) | TNM stage |
| --- | --- | --- |
| Case 1 | 56 | T_4a_N_0_M_0_ |
| Case 2 | 62 | T_4a_N_1a_M_0_ |
| Case 3 | 59 | T_4a_N_1a_M_0_ |
| Case 4 | 70 | T_4a_N_1b_M_0_ |
| Case 5 | 60 | T_4a_N_0_M_0_ |
| Case 6 | 72 | T_4a_N_1a_M_0_ |
| Case 7 | 57 | T_4a_N_1b_M_0_ |
| Case 8 | 71 | T_4a_N_1a_M_0_ |
| Case 9 | 66 | T_4a_N_1a_M_0_ |
| Case 10 | 83 | T_4a_N_1b_M_0_ |
